# Neurotoxicity of Immunotherapy: Immune Checkpoint Inhibitor-Related Encephalitis vs. Immune Effector Cell-Associated Neurotoxicity Syndrome

**DOI:** 10.14740/wjon2660

**Published:** 2025-12-17

**Authors:** Takumi Sato, Kohei Chida, Shipra Gandhi, Kazuaki Takabe

**Affiliations:** a Breast Surgery, Department of Surgical Oncology, Roswell Park Comprehensive Cancer Center, Buffalo, NY 14263, USA; b Department of Internal Medicine, Icahn School of Medicine at Mount Sinai, New York, NY, USA; c Department of Medicine, Winship Cancer Institute of Emory University, Atlanta, GA, USA; d Department of Immunology, Roswell Park Comprehensive Cancer Center, Buffalo, NY, USA; e Department of Surgery, University at Buffalo Jacobs School of Medicine and Biomedical Sciences, The State University of New York, Buffalo, NY 14263, USA; f Department of Breast Surgery and Oncology, Tokyo Medical University, Tokyo 160-8402, Japan; g Department of Gastroenterological Surgery, Yokohama City University Graduate School of Medicine, Yokohama 236-0004, Japan; h Division of Digestive and General Surgery, Niigata University Graduate School of Medical and Dental Sciences, Niigata 951-8520, Japan; i Department of Breast Surgery, Fukushima Medical University School of Medicine, Fukushima 960-1295, Japan

**Keywords:** Immune effector cell-associated neurotoxicity syndrome, Immune checkpoint inhibitor-related encephalitis, Immune-related adverse events, Chimeric antigen receptor T-cell, Bispecific T-cell engager, Cytokine release syndrome, Neurotoxicity, Immune checkpoint inhibitors

## Abstract

Immune checkpoint inhibitors and engineered T-cell therapies such as chimeric antigen receptor T-cell (CAR-T) cells and bispecific T-cell engagers (BiTEs) have revolutionized oncology care, and with them came two neurologic syndromes that look deceptively alike at the bedside with confusion, seizures, and encephalopathy: immune checkpoint inhibitor-related encephalitis (irEncephalitis) and immune effector cell-associated neurotoxicity syndrome (ICANS). Several differential observations between the two syndromes motivated this review: 1) although loss of immune tolerance likely drives irEncephalitis, ICANS on the other hand is dominated by cytokine-endothelial-microglial cascades. The biology of both entities remains incompletely resolved and these lines blur in real patients, 2) irEncephalitis is uncommon in ICI recipients (∼ 0.1-1%), whereas ICANS is common after CAR-T (∼ 40% and generally lower with most T-cell engagers), 3) lack of diagnostic and grading systems, especially the absence of a dedicated irEncephalitis grading system, remains the key barrier to consistent outcomes and meaningful comparison across clinical trials, and 4) management philosophies are asymmetric (restoring immune tolerance with selective immunomodulation in irEncephalitis vs. rapidly suppressing cytokine-mediated neuroinflammation with corticosteroids as well as anti-cytokine agents in ICANS). Here we review the existing literature on pathophysiology and current landscape of the diagnostics, management, and clinical trials to gain further structured understanding of these intriguing disorders. In doing so, we conclude that: 1) although the syndromes share similar clinical features, their pathogenesis points to distinct management algorithms based on timing of onset and response profiles, making mechanism-informed intervention central to improving outcomes; 2) early T-cell engager trials hint at molecule-dependent ICANS risk and responsiveness, warranting standardized reporting and accumulation of platform-specific data; and 3) emerging biomarkers and targets that index microglial signaling and blood-brain barrier integrity promise more precise, effective management as the field matures. In this review, we adopt a mechanism-first, side-by-side comparison that links diagnosis, management, and evolution to bedside decisions, with the aim of enabling precise diagnosis and management for oncologists, neurologists, and trialists operating in the rapidly expanding era of T-cell-based immunotherapy.

## Introduction

Immune checkpoint inhibitors (ICIs) have transformed cancer therapy by unleashing anti-tumor T-cell responses. Initially approved for melanoma, non-small cell lung cancer, and renal cell carcinoma, ICIs are now increasingly utilized in the more prevalent malignancies such as breast and gastrointestinal cancers [1-4]. However, their clinical efficacy has come at the cost of immune-related adverse events (irAEs), including a spectrum of neurologic toxicities. Among these, immune checkpoint inhibitor-related encephalitis (irEncephalitis), though relatively rare, represents a particularly serious and diagnostically challenging complication. The reported incidence of irEncephalitis varies between 0.1% and 1% among ICI-treated patients, depending on the study cohort and diagnostic criteria. A retrospective study of 1,228 ICI-treated patients found neurologic irAEs in 2.3% of cases, while other studies reported serious neurologic toxicities in 0.93-0.95% of patients [5-7]. Its presentation is often nonspecific, and its clinical course can be severe. Emerging evidence suggests that irEncephalitis may share mechanistic features with other forms of immune-mediated neurotoxicity, particularly involving T-cell overactivation, cytokine signaling, and central nervous system (CNS) inflammation; however, the pathogenesis of irEncephalitis remains relatively unclear.

Adoptive T-cell therapies, particularly chimeric antigen receptor T-cell (CAR-T) therapy, have also revolutionized the treatment of hematologic malignancies. Despite issues like poor infiltration, immunosuppressive tumor microenvironment (TME), and lack of tumor-specific antigens, numerous ongoing clinical trials are exploring CAR-T cell applications in various solid tumors, including glioblastoma, neuroblastoma, breast, colorectal, gastric, pancreatic, ovarian, and lung cancers [8-15]. Latest clinical strategies include dual-target CARs, use of variable domain of heavy-chain-only antibody (VHH)-based CARs (nanobody-based), and bispecific T-cell engager (BiTE), as well as novel technologies and engineering strategies such as CAR-NK, metabolic reprogramming, gene editing (CRISPR/Cas9), and combination therapies with checkpoint inhibitors or cytokines [16, 17]. As these platforms diversify, the choice of tumor target becomes increasingly critical. Multiple antigens are actively pursued, especially those associated with high expression levels in pro-inflammatory tumor microenvironment (TME). Emerging evidence indicates that tumor-intrinsic programs, such as reduced DNA repair capacity, can heighten treatment-responsive immunogenicity while at the same time prolonging inflammatory cascades [18-23]. Consistent with this concept, several targets correlated with TME immunogenicity have already been tested as CAR-T antigens in early clinical studies, showing signs of feasibility and therapeutic activity [24, 25].

Their success, however, is frequently accompanied with side effects, such as cytokine release syndrome (CRS) and immune effector cell-associated neurotoxicity syndrome (ICANS), two of the most prominent and potentially life-threatening irAEs in this context. Reported incidence rates of ICANS range widely depending on the product and study, but reach strikingly high levels in many trials compared to irEncephalitis. For instance, the pivotal CAR-T trial ZUMA-1 (axi-cabtagene ciloleucel in large B-cell lymphoma) reported neurologic adverse events in 64% of patients, with about 28% experiencing severe (grade ≥ 3) neurotoxicity [26]. Overall, across multiple CAR-T studies, all-grade ICANS has been observed in roughly 20-64% of patients, with severe ICANS in up to 50% in certain cohorts [27]. Similarly, T-cell engagers (TCEs) like blinatumomab show high neurotoxicity rates with clinical trials of blinatumomab in acute lymphoblastic leukemia reporting neurologic events in approximately 15-50% of patients [28]. Fortunately, most TCE-related neurotoxicities are low-grade (e.g. headache or tremor) and grade ≥ 3 neurologic events occur in roughly 5-15% of patients on blinatumomab, depending on the population [29]. While CRS has been increasingly well-characterized and effectively managed with targeted interventions such as interleukin-6 (IL-6) blockade, ICANS remains less understood with respect to its pathophysiology, diagnostic criteria, and optimal treatment strategies. However, despite differences in timing, underlying biology, and therapeutic management, irEncephalitis and ICANS share overlapping clinical features, including altered mental status, seizures, and encephalopathy. These observations raise a critical question: do irEncephalitis and ICANS represent entirely distinct entities, or do they both fall along a broader spectrum of immune-mediated encephalopathy? We aim to 1) summarize their clinical features, diagnostic approaches, and pathophysiologic underpinnings, 2) compare current grading systems and treatment algorithms, including the role of immunosuppressive agents such as corticosteroids and IL-6 blockade, and 3) summarize the current landscape of novel preclinical research and findings from early-stage clinical trials. By delineating their similarities and differences, we propose a framework for managing these conditions grounded in mechanistic understanding and one that is adaptable to the evolving landscape of T-cell-based immunotherapies.

## Neurologic irAEs: Focus on irEncephalitis and ICANS

### Etiologies: timing of onset and risk factors

irEncephalitis tends to develop within several weeks following ICI administration, with a reported median onset of approximately 5 weeks (mean 6.5 weeks) after initiation of ICIs [30, 31]. Several factors are thought to influence the likelihood of developing neurologic irAEs. Specific ICI regimens also appear to predispose to different neurologic phenotypes; for instance, anti-programmed cell death protein 1 (PD-1)/programmed cell death ligand 1 (PD-L1) therapy is more commonly associated with myasthenic syndromes, while anti-cytotoxic T-lymphocyte antigen-4 (CTLA-4) agents are linked to meningitis and cranial neuropathies [32]. Combination immune checkpoint blockade (e.g., CTLA-4 + PD-1 inhibitors) is associated with a significantly higher incidence of neurotoxicity (up to 13%) than monotherapy (3.8-6.1%) [33, 34]. Other reported risk factors include younger age, melanoma diagnosis, prior chemotherapy or surgery, and the presence of autoantibodies such as anti-Hu [35, 36]. Additionally, patients with pre-existing neurologic disorders, such as myasthenia gravis, multiple sclerosis, or autoimmune myositis, are at elevated risk of disease flare or worsening following ICI exposure; a relapse rate of up to 15.4% has been observed in such populations [37-39]. A systematic review of 83 patients who were rechallenged with ICIs after neurologic irAEs reported a recurrence rate of 19%, with no deaths attributed to recurrent neurologic toxicity, suggesting that rechallenge may be feasible in selected cases under careful monitoring [40].

In contrast, ICANS is far more common among patients receiving CAR-T cell therapy, occurring in approximately 40% [41]. Similar to irEncephalitis caused by ICIs, the type of costimulatory domain in CAR-T constructs has been shown to influence the timing and severity of ICANS. CAR-T therapies incorporating a CD28 costimulatory domain, such as axicabtagene ciloleucel (axi-cel), are associated with more rapid T-cell expansion, earlier onset, and greater severity of ICANS. In contrast, constructs with a 4-1BB domain, such as tisagenlecleucel (tisa-cel), promote more gradual T-cell activation and are linked to delayed onset and generally milder neurotoxicity [42]. Although ICANS typically occurs within days of CAR-T infusion, concurrently with or shortly after CRS, cases of “isolated ICANS”, neurotoxicity in the absence of CRS, have also been reported [43]. These cases may lead to delayed diagnosis and worse outcomes. Isolated ICANS is often associated with marked elevation of inflammatory cytokines and is thought to involve massive blood-brain barrier (BBB) disruption and endothelial activation as in CRS [44, 45]. Furthermore, in some patients, ICANS can follow a biphasic course, initial improvement followed by neurologic deterioration, or present as prolonged ICANS, with symptoms persisting beyond 3 weeks. These presentations appear to be more frequent among older adults and patients receiving CAR-T products with CD28 costimulatory domains [46, 47]. Additionally, patients who experience high-grade ICANS (≥ grade 3) are at greater risk of sustained neurologic deficits.

### Differences in pathophysiology of irEncephalitis and ICANS

irEncephalitis arises primarily from a loss of immune tolerance induced by ICIs (Table 1). These agents, by blocking inhibitory pathways, reinvigorate T-cell activity but can also provoke off-target immune responses against CNS tissues. This autoimmune assault results in inflammatory infiltration, neuronal damage, and in some cases the production of CNS-specific autoantibodies. Histopathological evidence supports this mechanism, such as cases showing dense CD8^+^ T-cell infiltrates in the meninges and parenchyma, alongside symptoms including cognitive impairment, seizures, and psychiatric disturbances [48, 49]. Beyond T-cell-mediated mechanisms, activation of resident glial cells such as astrocytes and microglia may amplify neuroinflammation in irEncephalitis, as evidenced by elevated cerebrospinal fluid (CSF) levels of glial fibrillary acidic protein (GFAP), S100B, and proinflammatory cytokines [50, 51]. Upstream inhibition of the Syk kinase has successfully attenuated this activation, highlighting its potential as a preventive or therapeutic strategy for ICI-associated neurotoxicity [52]. Astrocytes and microglia are known to upregulate PD-L1 expression in response to inflammatory stimuli and contribute to the regulation of neuroinflammation by inhibiting T-cell activation through the PD-1/PD-L1 axis. However, the use of ICIs may disrupt this regulatory mechanism, potentially leading to excessive immune responses within the CNS [53-55]. Furthermore, B-cell and antibody-mediated pathways are also known to play a crucial role. For example, a 2025 case series by Buckley et al reported 14 patients with ICI-related encephalitis - about 31% of these patients had identifiable neural autoantibodies in serum or CSF [56, 57]. Detected antibodies in that cohort ranged from neural surface proteins (like CASPR2/LGI1 and N-methyl-D-aspartate (NMDA) receptors) to classic paraneoplastic onconeural antigens (e.g. anti-Hu, anti-Ma2) [58, 59]. Mechanistically, checkpoint inhibitors can dysregulate B-cell tolerance and promote autoantibody formation by directly modulating B-cell function as well as causing an expansion of CD21-low B cells, with pronounced changes in the setting of combined uses [60].

**Table 1:** Comparison Between irEncephalitis and ICANS

	irEncephalitis	ICANS
Pathogenesis	Autoimmune (T-cell or autoantibody-mediated)	Cytokine-mediated inflammation post-CAR-T therapy
First-line therapy	Corticosteroids	Corticosteroids (ICANS ≥ grade 2); tocilizumab (with CRS)
Adjuncts	IVIG, PLEX, rituximab (autoantibody-driven cases)	Tocilizumab, anakinra (off-label), anti-epileptics
Immune checkpoint use	Discontinue or permanently hold ICIs	Continue CAR-T while managing toxicity if possible
Monitoring	MRI, CSF, autoantibodies	ICE score, cytokine levels, GFAP/NFL in CSF

Comparison of pathogenesis, first-line therapy, adjunctive treatments, immune checkpoint inhibitor usage, and monitoring strategies between irEncephalitis and ICANS. CAR-T: chimeric antigen receptor T-cell; CRS: cytokine release syndrome; CSF: cerebrospinal fluid; GFAP: glial fibrillary acidic protein; ICANS: immune effector cell-associated neurotoxicity syndrome; ICE: immune effector cell-associated encephalopathy; ICI: immune checkpoint inhibitor; irEncephalitis: immune-related encephalitis; IVIG: intravenous immunoglobulin; MRI: magnetic resonance imaging; NFL: neurofilament light chain; PLEX: plasma exchange.

ICANS emerges within the first few days following CAR-T infusion, frequently coinciding with or following CRS, while irEncephalitis typically develops subacutely, often weeks to several months after initiation of immune checkpoint blockade. The massive expansion and activation of CAR-T cells trigger systemic release of inflammatory cytokines (IL-6, interferon (IFN)-γ, granulocyte-macrophage colony-stimulating factor (GM-CSF), etc.), leading to disruption of the BBB, endothelial activation, and microglial inflammation [61]. BBB disruption permits inflammatory mediators and immune cells to enter the CNS, where they induce neurotoxicity. Notably, myeloid cells play a critical role in this process. *In vitro* co-culture experiments have shown that CAR-T cells trigger monocytes to release IL-6 and other cytokines, and this process depends on GM-CSF signaling, therefore functioning as a pivotal paracrine signal. Studies have revealed that IL-1β compromises BBB integrity by disrupting astrocyte-endothelial interactions. Specifically, IL-1β suppresses the sonic hedgehog (SHH) signaling pathway in astrocytes, which is normally crucial for maintaining endothelial tight junctions. This allows more immune cells to enter CNS, eventually forming feed-forward loop (CAR-T → GM-CSF → monocyte IL-1/IL-6 → endothelial activation → BBB leak → more CNS immune infiltration) [62]. Indeed, preclinical models have demonstrated that inhibition of GM-CSF, a key myeloid activation factor, significantly ameliorates both CRS and ICANS severity, highlighting the pivotal contribution of myeloid-driven inflammation to its pathogenesis and its potential as target for treatment [63]. Elevated levels of GFAP and neurofilament light chain (NFL) in CSF reflect astrocytic and axonal injury, respectively [64, 65]. The elevation of the serum angiopoietin-2 (ANG2) to angiopoietin-1 (ANG1) ratio in patients with ICANS has been reported to be associated with endothelial activation, disruption of the BBB, and coagulation abnormalities [66-68]. Emerging studies have identified transforming growth factor (TGF)-β-activated kinase-1 (TAK1)-mediated mitogen-activated protein kinase (MAPK) signaling and microglial activation as central contributors to ICANS, suggesting new aspects of pathogenesis as well as therapeutic targets for intervention [26, 52].

### Diagnosis: key symptoms, imaging, and laboratory findings

irEncephalitis often presents with hyperintense lesions on magnetic resonance imaging (MRI) fluid-attenuated inversion recovery (FLAIR) sequences, typically in the limbic system but potentially also in extralimbic or meningeal regions. CSF frequently reveals lymphocytic pleocytosis and elevated protein levels [69]. 18F-FDG positron emission tomography (PET) imaging has shown high diagnostic yield (sensitivity ∼ 87%) for encephalitis caused by autoantibodies, and can sometimes detect abnormalities not visible on MRI, as suggested by a meta-analysis performed by Bordonne et al [70]. They also suggested that different antibody subtypes can produce distinct metabolic patterns on PET scans, underscoring the importance of this modality in the management of irEncephalitis. While the above features support irEncephalitis, a structured differential is essential in cancer patients and must explicitly weigh common mimics. Key differentials and distinguishing clues are as follows. 1) Infectious encephalitis often presents with fever, early neutrophilic CSF, and herpes simplex virus (HSV)-predominant temporal-lobe diffusion-weighted imaging (DWI) changes with PCR positivity [71, 72]. 2) Autoimmune encephalitis includes paraneoplastic, onconeural-antibody syndromes (e.g., anti-Hu/Ma2; frequently steroid-refractory) and surface-antigen disorders (e.g., NMDAR, LGI1 with faciobrachial dystonic seizures; usually responsive to immune modulation) [73, 74]. 3) Brain metastases or leptomeningeal disease manifest with nodular parenchymal or linear leptomeningeal enhancement and malignant cells on CSF cytology when positive [75]. 4) Metabolic/toxic encephalopathy is suggested by fluctuating attention, asterixis/myoclonus, triphasic electroencephalograph (EEG) waves, normal CSF, and drug triggers (e.g., opioids, cefepime, calcineurin inhibitors) [76]. 5) Stroke or post-ictal states show vascular-territory DWI restriction or transient post-ictal deficits (e.g., Todd’s paresis) and often non-territorial, reversible peri-ictal MRI changes. A pragmatic approach is to exclude infection first, then integrate MRI with DWI/contrast or FDG-PET if MRI remains non-diagnostic but suspicion is high, EEG, comprehensive CSF studies (including viral PCR and cytology), and a broad neuronal-antibody panel.

Early recognition of CRS is crucial for diagnosing ICANS. CRS can present with a wide spectrum of clinical manifestations, and should be considered early in the differential diagnosis [77]. Mild CRS may initially present with nonspecific symptoms such as fever, fatigue, headache, rash, arthralgia, myalgia, cough, and tachypnea. However, progression to severe disease can involve hypotension, high-grade fever, vascular leakage, disseminated intravascular coagulation (DIC), and multi-organ dysfunction syndrome [78]. Notably, a large proportion of severe CRS cases fulfill clinical criteria for sepsis, and many even meet criteria for septic shock, making differentiation extremely challenging. Notably, laboratory abnormalities can aid in early recognition of CRS. Five key abnormalities commonly observed include: 1) cytopenias, 2) elevated serum creatinine, 3) elevated liver transaminases, 4) coagulation abnormalities, and 5) elevated C-reactive protein (CRP) levels. Awareness of these laboratory features is critical to prompt diagnosis and management.

However, the onset of neurologic symptoms does not always coincide with the development of CRS. As discussed earlier, neurologic symptoms can occur before, during, or even after the resolution of CRS, sometimes presenting as biphasic or isolated patterns, adding an additional layer of complexity to diagnosis. Additionally, the clinical manifestations of ICANS are broad, ranging from mild confusion with expressive aphasia, headache, and hallucinations, to more severe presentations such as global aphasia, hemiparesis, cranial nerve palsies, seizures, and somnolence. Moreover, ICANS frequently presents with normal MRI findings, although subtle changes such as cerebral edema may be observed in severe cases [41, 61]. CSF findings include elevated protein with mild pleocytosis, while biomarkers such as GFAP and NFL in CSF and serum serve as indicators of astrocytic and axonal injury, respectively [64, 65]. Cytokine profiling - especially levels of IL-6, IL-1, IFN-γ, and GM-CSF in blood and CSF - can aid in evaluating the inflammatory milieu and severity of ICANS [64]. The immune effector cell-associated encephalopathy (ICE) score is a key clinical tool used to monitor cognitive and neurological function in these patients. While the above features support ICANS, a structured differential is essential in recipients of immune-effector cell therapies. Common mimics and distinguishing clues include: 1) Sepsis-associated encephalopathy or systemic infection can manifest with fever/shock, metabolic derangements, and diffuse EEG slowing; note that severe CRS frequently fulfills sepsis or even septic-shock criteria, making early cultures and antimicrobials critical [78, 79]. 2) CNS infection can present with headache/meningismus and can be evaluated by early neutrophilic CSF and pathogen-specific polymerase chain reaction (PCR) (e.g., HSV), with repeat testing when early false negatives are suspected [72]. 3) Acute cerebrovascular events (ischemic/hemorrhagic stroke) characterized by hyperacute focal deficits with vascular-territory DWI restriction or acute hemorrhage on computed tomography (CT)/MRI. 4) Posterior reversible encephalopathy syndrome (PRES) can mimic ICANS, presenting with headache, visual symptoms, seizures with posterior-predominant vasogenic edema on FLAIR, often in the setting of hypertension or calcineurin-inhibitor exposure [80]. 5) The pretest probability of CNS infection in recipients of CAR-T and BiTE can be high and the presentations can mimic ICANS, and, most importantly, may alter early management. A single-cell/viral-genomics work showed that latent human herpesvirus (HHV)-6B may reactivate within CAR-T cells during manufacturing and activation, so early plasma and CSF HHV-6 PCR should be considered when altered mental status (AMS) or seizures emerge post-infusion [81]. In large cohorts, 6% plasma HHV-6B reactivation and 0.2% possible HHV-6 encephalitis within 12 weeks post-CAR-T have been reported [82]. Moreover, in patients with an Ommaya reservoir, which is not infrequently present in this population, device-associated ventriculitis or meningitis remains an important differential, with ∼ 5-8% infection rates across large series, arguing against steroid-first approached before appropriate diagnostic evaluations [83, 84]. A pragmatic workup is to exclude infection first, then integrate CT/MRI with DWI (also consider magnetic resonance arteriography (MRV)), EEG, and comprehensive CSF studies (cell count, chemistries, viral PCR, cytology).

### Comparison with other types of neurological immune events: ICI-induced meningitis

For the sake of comparison, we shall briefly discuss pathophysiology, management, and prognosis of meningitis caused by ICIs. ICI-induced meningitis is less common compared to other neurologic toxicities such as myositis and encephalitis. It is often associated with anti- CTLA-4 inhibitors rather than anti- PD-1 inhibitors [38]. Similar to encephalitis, the pathophysiology of irMeningitis is speculated to involve autoimmune response, cytokine release, and disruption of the BBB. However, other pathways that involve mechanisms similar to CRS are indeed speculated to play a role, suggested by a combined case of hemophagocytic lymphohistiocytosis and meningitis due to atezolizumab treatment [85]. Mechanistically, irEncephalitis is largely autoimmunity/tolerance-loss with CSF lymphocytic pleocytosis and FLAIR lesions, while ICANS reflects cytokine-endothelial-microglial cascades with frequently normal MRI but elevated protein ± mild pleocytosis. In comparison, irMeningitis shares autoimmune/inflammatory meningeal involvement and rarely shows CRS-like features as described in hemophagocytic lymphohistiocytosis (HLH)-plus-meningitis.

The median time to onset of meningitis symptoms post-ICI administration varies, with some studies reporting a median of 9 days and others indicating a median of 21 days [86, 87]. By comparison, irEncephalitis usually presents subacutely about 5 weeks after ICI initiation, whereas ICANS typically arises within days of immune-effector cell infusion and often coincides with or follows CRS (see Sections “Etiologies: timing of onset and risk factors” to “Diagnosis: key symptoms, imaging, and laboratory findings”). This “earlier-than-encephalitis yet later-than-ICANS” timing helps triage workup when new headache/meningismus appears after starting immunotherapy.

Although direct head-to-head data are limited, ICI-associated meningitis is typically steroid-responsive with low short-term mortality and limited persistent deficits [87-90]. In a case series, four out of seven patients with ICI-related meningitis were treated with steroids, resulting in complete recovery within 2 weeks. The remaining patients improved spontaneously within 3 weeks [87]. This suggests that while steroids are beneficial, some cases may resolve without them, while in irEncephalitis, escalation beyond high-dose corticosteroids (intravenous immunoglobulin (IVIG)/plasma exchange (PLEX)/rituximab) is not infrequent and ICANS is managed by the American Society for Transplantation and Cellular Therapy (ASTCT) grade with dexamethasone and anti-cytokine strategies in the presence of CRS.

The prognosis for patients with ICI-related meningitis is generally favorable. In the case series described above, all patients who experienced meningitis were able to resume ICI therapy without recurrence of meningitis, indicating that rechallenge with ICIs can be safe under careful monitoring [87]. This is an important comparison to ICANS where cellular products are not “stopped” *per se* (CAR-T is single-infusion; TCEs use dose holds) or irEncephalitis which typically prompts ICI interruption and individualized rechallenge.

Taken together, ICI-induced meningitis appears to have a more favorable clinical trajectory than irEncephalitis or ICANS, with earlier symptom onset, higher rates of spontaneous or steroid-responsive recovery, and a lower risk of treatment-limiting recurrence. These features highlight that not all immune-mediated neurological adverse events carry the same risk profile. Therefore, distinguishing meningitis from encephalitis in the clinical setting is crucial not only for prognostication but also for guiding therapeutic decisions, including the safe resumption of immunotherapy under careful surveillance.

## Comparison of Management and Therapeutic Strategies

### Grading systems

Grading the severity of neurologic toxicity is essential for standardized management and early therapeutic intervention. For irEncephalitis, severity is typically assessed based on clinical judgment, guided by American Society of Clinical Oncology (ASCO) and National Comprehensive Cancer Network (NCCN) recommendations [91]. These frameworks incorporate symptom intensity, imaging findings, and functional impairment, and help determine when to withhold ICIs or escalate immunosuppression. However, a universally accepted grading scale specific to irEncephalitis is still lacking, in part due to its low incidence and clinical heterogeneity [30].

In contrast, ICANS is evaluated using the standardized grading system developed by the ASTCT, which integrates the ICE score, level of consciousness, presence of seizures, motor deficits, and signs of cerebral edema (Tables 2 and 3). This system enables clinicians to triage patients and implement stage-specific treatments such as corticosteroids or IL-6 blockade (e.g., tocilizumab) in CRS-associated cases. It also facilitates the coordination of neurologic and systemic management in overlapping CRS/ICANS presentations. However, it is important to note that the ICANS grading system was originally developed in the context of CAR-T therapy, and its applicability to other treatments such as TCEs remains a subject of ongoing discussion.

**Table 2:** ICE Score

Neurologic status	Grade 1	Grade 2	Grade 3	Grade 4
ICE score	7 - 9	3 - 6	1 - 2	0
Consciousness	Spontaneously awake	Awakens to voice	Awakens to tactile stimulus	Unresponsive to all stimuli, coma or obtundation
Seizure	N/A	N/A	Seizure ≤ 5 min	Seizure > 5 min or intractable
Motor findings	N/A	N/A	N/A	Severe hemiparesis or global paresis
ICP/cerebral edema	N/A	N/A	Focal edema	Diffuse cerebral edema, cranial nerve palsy, brainstem edema, Cushing’s triad

ASTCT ICE scoring system for ICANS, detailing neurologic status and associated clinical findings across severity grades. ASTCT: American Society for Transplantation and Cellular Therapy; ICANS: immune effector cell-associated neurotoxicity syndrome; ICE: immune effector cell-associated encephalopathy; ICP: intracranial pressure.

**Table 3:** Grading and Treatment of ICANS

ASTCT grade	Without CRS	With CRS
Grade 1	Supportive care only	Tocilizumab 8 mg/kg (max 800 mg) IV over 1 h, repeat per ASTCT/institutional protocol when CRS persists (observe maximum total dosing limits)
Grade 2	Dexamethasone 10 mg IV, repeat every 6 - 12 h if needed	As in grade 1 + ICU if CRS ≥ grade 2
Grade 3	ICU care, dexamethasone 10 mg q6h or methylprednisolone 1 mg/kg q12h, consider imaging every 2 - 3 days	Same as grade 1
Grade 4	ICU and respiratory support, methylprednisolone 1,000 mg/day for 3 days then taper, consider anakinra 100 mg q12h if steroid-resistant	Same as grade 1

Management algorithms for ICANS according to ASTCT grade, stratified by the presence or absence of concomitant CRS, highlighting escalation steps from supportive care to intensive immunosuppressive therapies. ASTCT: American Society for Transplantation and Cellular Therapy; CRS: cytokine release syndrome; ICANS: immune effector cell-associated neurotoxicity syndrome; ICU: intensive care unit; IV: intravenous.

### Treatment of irEncephalitis

Management of irEncephalitis centers on suppressing the autoimmune inflammatory response triggered by ICIs (Table 4). The first step is prompt discontinuation of ICIs, followed by immunosuppressive therapy. High-dose corticosteroids such as methylprednisolone are typically the first-line treatment [59]. In patients with inadequate response or severe presentations, adjunctive therapies including IVIG, PLEX, and rituximab may be considered, particularly when neuronal autoantibodies such as anti-Hu or anti-Ma2 are detected [30]. Indeed, the presence of specific autoantibodies may also influence therapeutic decisions. For instance, cases associated with anti-glutamic acid decarboxylase (GAD) or anti-GFAP antibodies often show good steroid responsiveness, whereas those with onconeuronal antibodies (e.g., anti-Hu) may require more aggressive treatment due to a poorer prognosis [92]. There is growing interest in precision immunomodulation targeting cytokine pathways or specific immune cells in cancer treatment, but evidence remains limited. Therefore, current practice is largely based on expert consensus and observational studies.

**Table 4:** Clinical Grading and Corresponding Treatment Recommendations for irEncephalitis

Grade	Clinical features	Treatment strategy
Grade 1	Mild symptoms (e.g., headache, low-grade fever, mild cognitive changes), stable neurological exam	Hold ICIs, initiate oral corticosteroids (e.g., prednisone 0.5 - 1 mg/kg/day), close monitoring
Grade 2	Moderate symptoms (e.g., confusion, disorientation), abnormal findings on MRI or CSF, no severe functional impairment	Discontinue ICIs, high-dose IV corticosteroids (e.g., methylprednisolone 1 - 2 mg/kg/day), neurology consult
Grade 3	Severe symptoms (e.g., seizures, focal neurologic deficits), evidence of CNS inflammation, positive autoantibodies	Escalate to IVIG or plasmapheresis if refractory; consider rituximab or mycophenolate depending on antibody profile
Grade 4	Life-threatening symptoms (e.g., status epilepticus, coma), diffuse brain involvement, ICU-level care	ICU care, aggressive immunosuppression (methylprednisolone pulse 1,000 mg/day for 3 - 5 days), consider cyclophosphamide

Clinical grading and corresponding treatment recommendations for irEncephalitis, based on symptom severity, imaging findings, CSF abnormalities, and presence of autoantibodies. CSF: cerebrospinal fluid; ICI: immune checkpoint inhibitor; ICU: intensive care unit; irEncephalitis: immune-related encephalitis; IVIG: intravenous immunoglobulin; MRI: magnetic resonance imaging.

### Treatment of ICANS

In contrast, the treatment of ICANS emphasizes control of cytokine-mediated neuroinflammation (Table 3). Corticosteroids are the cornerstone of ICANS management, particularly for grade 2 or higher toxicity according to ASTCT criteria [91]. Dexamethasone is commonly used due to its CNS penetration and anti-inflammatory potency. For ICANS associated with CRS, tocilizumab, an IL-6 receptor antagonist, is recommended as first-line therapy. While tocilizumab is approved for CRS, it is important to note that it may not cross the BBB effectively, resulting in limited efficacy for ICANS symptoms. Nonetheless, it plays a crucial role in reducing systemic inflammation and may indirectly benefit neurologic symptoms [93].

While current ICANS grading and management frameworks were originally developed for CAR-T therapies, emerging evidence suggests their applicability to multiple TCE platforms as well [94]. Across TCE platforms, a common pattern has been observed in which CRS precedes the onset of neurotoxicity, and the improvement of CRS seems to lead to the secondary improvement of neurologic symptoms caused by other TCEs. However, dedicated toxicity characterization in TCE trials remains limited, and future guidelines should address the nuances of TCE-specific presentations.

In patients with refractory or recurrent ICANS, anakinra, an IL-1 receptor antagonist, has been explored as a potential alternative to tocilizumab, as IL-1 is speculated to play a central role and experiments in murine models revealed that its inhibition reduces neuronal toxicity of immune therapy [95]. According to a recent update of ASCO Clinical Practice Guidelines, anakinra is not FDA-approved for CRS, and its use remains off-label [96]. Key considerations include contraindication to co-administration with tumor necrosis factor (TNF)-α inhibitors, risks in the context of active or chronic infections and uncertain long-term malignancy risk, need for neutrophil count monitoring, and avoidance of concurrent administration with live vaccines. Despite these cautions, there are accumulating case reports and small series suggesting anakinra’s benefit in severe or steroid-refractory ICANS and CRS, particularly given its CNS penetration and role in modulating IL-1-mediated microglial activation [97].

Antiepileptic drugs such as levetiracetam are frequently administered for seizure prophylaxis or treatment, especially in severe ICANS. Their use is supported by the high incidence of subclinical seizures and the potential for status epilepticus in advanced cases [98].

While off-label, recent studies have also documented the expanding use of tocilizumab in severe or steroid-refractory ICANS in other T-cell-engaging therapies, including tarlatamab, a delta-like ligand 3 (DLL3)-targeting BiTE. Blinatumomab has been reported to cause CRS in approximately 40-50% of patients, and ICANS-like neurotoxicity in 10-15% [99]. Clinical trial data show that patients receiving tarlatamab who developed CRS or neurotoxicity were successfully managed with tocilizumab, reinforcing its central role across different immunotherapeutic platforms. In the DeLLphi-300 trial evaluating tarlatamab, tocilizumab effectively managed grade ≥ 2 CRS and was also associated with improvement in associated neurologic events. These findings suggest that anakinra use, now off-label, can be considered, supported by its CNS penetration and biologic rationale for IL-1-mediated microglial activation.

In summary, ICANS is an anticipated, generally time-limited toxicity of immune-effector cell therapies associated with cytokine-driven endothelial/microglial activation. It is graded and treated algorithmically (ASTCT/ICE) with short-course corticosteroids, prompt anti-cytokine therapy when CRS is present (e.g., tocilizumab), and currently off-label use of anakinra considered for steroid-refractory courses. By contrast, irEncephalitis is a rare, autoimmune loss-of-tolerance toxicity in which clinicians should maintain a low threshold to hold ICIs, pursue CSF/MRI/antibody workup, and escalate early to multimodal immunosuppression (high-dose steroids → IVIG/PLEX ± B-cell-directed agents) when recovery is incomplete. Operationally, timing relative to therapy (days for ICANS vs. weeks for irEncephalitis), typical investigations (ICANS with frequently normal MRI and irEncephalitis with lymphocytic CSF associated with limbic/extralimbic FLAIR abnormalities), and the risk of residual neurologic impairment (higher in irEncephalitis) guide treatment intensity and decisions around therapy interruption or rechallenge. These distinctions underscore a mechanism-oriented, team-based approach of each syndrome rather than phenotype (Tables 2-4).

## Future Directions

Although irEncephalitis and ICANS share overlapping clinical features such as confusion, seizures, and encephalopathy, their underlying pathogenesis demands distinct therapeutic approaches. Ultimately, mechanism-informed intervention is the key to improving outcomes. While irEncephalitis requires modulation of autoimmune processes, ICANS demands rapid suppression of cytokine-driven neuroinflammation and stabilization of CNS homeostasis. Novel biomarkers and therapeutic targets (e.g., microglial signaling, BBB integrity) are under investigation and hold promise for more precise and effective management in the future [96, 100].

The landscape of TCE therapies is rapidly evolving. As of November 2023, seven TCE therapies have received clinical approval, reflecting significant advancements in this field [52, 101]. There has been a surge in the number of clinical trials investigating TCEs, with over 600 trials reported in the past year alone. Notably, three TCEs received approval in the latter half of 2024, highlighting the accelerated pace of development and regulatory endorsement in this domain. With their rapid growth, reports on ICANS with the use of newer T-cell engagement modalities are being accumulated (Table 5 [102-110]). While the overall incidence of ICANS appears lower than that observed with CAR-T therapies - typically less than 5% for grade ≥ 3 events across most agents - blinatumomab, a CD19 × CD3 BiTE, remains a notable exception. In clinical trials, grade ≥ 3 neurotoxicity has been observed in approximately 7-15% of patients, with seizures occurring in 1-2%. This increased risk may be partially explained by the expression of CD19 on perivascular mural cells within the CNS [111]. Importantly, most ICANS events associated with blinatumomab are reversible and tend to occur during the initial treatment cycle, typically resolving within 3 to 5 days [102, 112]. Reports suggest that 9% of patients taking talquetamab experience ICANS (all-grade) and 6% grade ≥ 3 neurologic adverse events (no grade 4 ICANS); however, most neurotoxicities were headache or encephalopathy.

**Table 5:** Incidence of ICANS in Key T-Cell Engaging Therapies (Phase II or Later)

Therapy (type)	Targets (tumor × T-cell)	Indication (tumor type)	Status	ICANS incidence (any; grade ≥ 3)
Blinatumomab (BiTE)	CD19 × CD3	B-ALL (RR)	FDA-approved	57% any neurotoxicity; ∼ 11-13% grade ≥ 3 ICANS [102, 103]
Teclistamab (BsAb)	BCMA × CD3	RRMM	FDA-approved	6% any ICANS; ∼ 2% grade ≥ 3 ICANS [104]
Talquetamab (BsAb)	GPRC5D × CD3	RRMM	FDA-approved	9% any ICANS; ∼ 6% grade ≥ 3 neurologic toxicity
Elranatamab (BsAb)	BCMA × CD3	RRMM	FDA-approved	∼ 3-4% any ICANS; 0% grade ≥ 3 ICANS reported [105]
Epcoritamab (BsAb)	CD20 × CD3	3L+ R/R LBCL	FDA-approved	6% any ICANS (all grade 1-2); ∼ 0% grade ≥ 3 (one fatal ICANS ∼ 0.6%) [106]
Glofitamab (BsAb)	CD20 × CD3	R/R DLBCL	FDA-approved	4.8% any ICANS; ∼ 0% grade ≥ 3 ICANS (2.1% grade ≥ 3 neuro events) [107]
Mosunetuzumab (BsAb)	CD20 × CD3	R/R FL, 3L+	FDA-approved	∼ 1% any ICANS (grade 1-2 only); 0% grade ≥ 3 ICANS [108]
Odronextamab (BsAb)	CD20 × CD3	B-cell NHL (R/R DLBCL, FL - phase II ELM-2 trial)	Phase II	0% ICANS reported (none observed in trials) [109]
Tebentafusp (TCR-CD3)	gp100-HLA*A2:01 × CD3	Uveal melanoma (metastatic)	FDA-approved	< 1% ICANS (CRS common; no significant ICANS reported) [110]

ICANS incidence for prominent T-cell engager therapies (including bispecific T-cell engagers, bispecific antibodies, and TCR-mimic constructs) that are approved or in phase II/III development. Both overall ICANS frequency (any grade) and the incidence of high-grade (≥ 3) ICANS are provided where available, along with each agent’s targets, cancer indication, regulatory status. B-ALL: B-cell acute lymphoblastic leukemia; CRS: cytokine release syndrome; DLBCL: diffuse large B-cell lymphoma; FL: follicular lymphoma; ICANS: immune effector cell-associated neurotoxicity syndrome; LBCL: large B-cell lymphoma; MM: multiple myeloma; NHL: non-Hodgkin lymphoma; RR: relapsed/refractory.

Emerging evidence suggests that incorporating a third specificity, particularly targeting co-stimulatory molecules such as CD28, 4-1BB, or OX40, in addition to CD3 on T cells and a tumor-associated antigen (TAA) on cancer cells, can enhance T-cell activation and prevent exhaustion. This platform offers modularity, allowing for the exchange of different single-chain fragment variable (scFv) fragments to target various TAAs, and has demonstrated superior antitumor activity and T-cell proliferation in preclinical models. However, the challenge of adverse events still remains; certain TCEs targeting specific TAAs or with a CD3 binding domain with weaker affinity have been linked to unique toxicity profiles, such as hepatotoxicity or dermatologic reactions [113]. In a phase I trial involving patients with relapsed or refractory B-cell malignancies, CC312 (CD19 × CD3 × CD28) demonstrated favorable tolerability, with no grade ≥ 3 ICANS reported [114]. However, the available data remain limited, and continued observation will be essential to fully understand the safety profile of this modality. Understanding these nuances is crucial for developing tailored management strategies and informing clinical trial designs.

This review highlights the importance of distinguishing autoimmune-driven irEncephalitis from cytokine-mediated ICANS, and calls for standardized, pathology-informed treatment algorithms. Future studies should incorporate these frameworks into real-world clinical pathways to improve patient safety and therapeutic outcomes.

## Conclusions

irEncephalitis and ICANS often share overlapping clinical features but arise from distinct mechanisms, making it essential to distinguish between them and to rule out common mimics before initiating treatment. Mechanism-guided interventions remain central to outcomes: restoring immune tolerance in irEncephalitis versus rapidly suppressing cytokine-driven inflammation in ICANS. Standardized grading, platform-specific reporting and data from early T-cell engager trials, together with emerging biomarkers of microglial activation and BBB integrity, will be key to advancing precise management.

## Data Availability

Data sharing is not applicable to this article as no datasets were generated or analyzed during the current study.

## Abbreviations

BiTE: bispecific T-cell engager
CAR-T: chimeric antigen receptor T-cell
CRS: cytokine release syndrome
GFAP: glial fibrillary acidic protein
ICANS: immune effector cell-associated neurotoxicity syndrome
ICE: immune effector cell-associated encephalopathy
ICI: immune checkpoint inhibitor
irAE: immune-related adverse event
irEncephalitis: immune checkpoint inhibitor-related encephalitis
NFL: neurofilament light chain
PRES: posterior reversible encephalopathy syndrome
TAA: tumor-associated antigen
TAK1: transforming growth factor beta-activated kinase 1
TCE: T-cell engager
TME: tumor microenvironment
